# Multiple Shorter High‐Intensity Interval Exercise Sessions During the Day Result in Greater Energy Expenditure With Less Exertion Than a Longer Single Session: A Randomized Crossover Clinical Trial

**DOI:** 10.1002/ejsc.12302

**Published:** 2025-05-02

**Authors:** Gilton de Jesus Gomes, Caíque Olegário Diniz Magalhães, Ilkilene Pinheiro Queiroz, Júllia Alves de Andrade, Bruna Caroline Chaves Garcia, Ramona Ramalho de Souza Pereira, Joyce Mirlane Moreira Costa, Ricardo Cardoso Cassilhas, Daniel Campos Villela, Flávio Castro de Magalhães, Fernando Gripp, Fabiano Trigueiro Amorim, Marco Fabrício Dias‐Peixoto

**Affiliations:** ^1^ Department of Physical Education Federal University of the Jequitinhonha and Mucuri Valleys (UFVJM) Diamantina Brazil; ^2^ Multicenter Graduate Program in Physiological Sciences Brazilian Society of Physiology Diamantina Brazil; ^3^ Graduate Program in Health Sciences Federal University of the Jequitinhonha and Mucuri Valleys (UFVJM) Diamantina Brazil; ^4^ Department of Health, Exercise, and Sports Sciences University of New Mexico Albuquerque New Mexico USA

**Keywords:** accumulated exercise, excess post‐exercise oxygen consumption, exercise snacks, high‐intensity interval exercise

## Abstract

Breaking up periods of sedentary time with brief bouts of high‐intensity interval exercise (HIIE) is suggested as a time‐efficient approach to improve exercise adherence and health. This randomized crossover clinical trial was designed to compare energy expenditure (EE), cardiometabolic, and perceptual responses during HIIE performed in a single session (1xHIIE) or split into three shorter sessions (3xHIIE) throughout the day. Fifteen male participants (48.5 ± 2.9 years) completed two experimental protocols: 1xHIIE protocol consisted of a single 21 min session, whereas the 3xHIIE protocol consisted of three shorter 7 min sessions separated by a 4 h interval between each session. Oxygen consumption (VO_2_), heart rate (HR), rate of perceived exertion (RPE), and blood lactate were measured during the experimental protocols. The 1xHIIE and 3x HIIE sessions induced similar EE (298.20 ± 51.74 and 299.32 ± 69.18 kcal, respectively; *p* = 0.88). However, postexercise EE following the 3xHIIE was approximately twice as high as the 1xHIIE (62.97 ± 14.97 vs. 27.42 ± 8.98 kcal, respectively; *p* < 0.001) or approximately 36 kcal higher. Additionally, compared to 1xHIIE, the 3x HIIE protocol induced lower HR (158 ± 12 and 147 ± 8 bpm, respectively; *p* = 0.018), rating perceived effort (15.8 ± 1.8 and 14.4 ± 1.7 respectively; *p* = 0.0012), and blood lactate (7.7 ± 3.7 and 5.4 ± 1.8 mmol/L, respectively; *p* = 0.013). These findings suggest that multiple brief sessions of HIIE throughout the day result in a greater energy expenditure with less perceived exertion than a single HIIE session in middle‐aged male individuals.

**Summary**

- Exercise snacks are an effective way to increase energy expenditure.
- Exercise snacks require a lower effort.
- Exercise snacks offer a viable alternative to improve adherence to physical exercise.

## 1 Introduction

Physical inactivity is considered as the fourth leading risk factor for global mortality (6% of deaths globally) accounting for approximately 3.2 million deaths per year (WHO 2009). More than a quarter of the world's adult population (1.4 billion adults) are physically inactive and not meeting the current recommendations of at least 150 min of moderate‐intensity or 75 min of vigorous‐intensity physical activity per week (Hallal et al. 2012; WHO 2022). Usually, this lack of physical activity is associated with sedentary behavior, defined as waking behavior characterized by an energy expenditure ≤ 1.5 metabolic equivalents (METs). Interestingly, individuals who meet the current recommendation for physical activity but spend prolonged hours in sedentary behavior, such as prolonged sitting in occupations or during leisure time, may still be susceptible to cardiovascular and metabolic diseases (Lavie et al. 2019). Therefore, it becomes crucial to identify exercise strategies that can interrupt sedentary behavior even in physically active individuals, aiming to reduce the risk of cardiovascular and metabolic diseases.

Breaking up exercise into short bouts (< 10 min) throughout the day could be more manageable for most individuals and serve as a feasible strategy to integrate into a daily routine, whether at home or even in the workplace (Gillen et al. 2021; Broadney et al. 2018). An effective approach to breaking up sedentary behavior and improving cardiometabolic health in physically inactive individuals is by using short‐duration bouts of vigorous exercise, such as high‐intensity interval exercise (HIIE), throughout the day, commonly known as “exercise snacks” (Islam et al. 2022). Previous studies have demonstrated that these short‐duration bouts of vigorous exercise distributed throughout the day may be more effective in improving cardiometabolic markers than a single prolonged session of moderate or vigorous exercise. For instance, Francois (Francois et al. 2014) demonstrated that a brief intense exercise bout before each main meal were more effective in reducing postprandial and 24 h mean blood glucose concentration than a single session of prolonged continuous exercise in individuals with insulin resistance. In an experimental animal model, we demonstrated that 8 weeks of a high‐intensity interval training protocol with three daily HIIE sessions was superior to a single HIIE daily session with similar exercise volume and intensity in reducing visceral fat weight and adipocyte size (Mendes et al. 2022). This superiority was observed irrespective of energy intake, and we speculate that multiple daily short‐duration sessions might result in higher energy expenditure (EE) than single daily sessions due to higher accumulated excess postexercise oxygen consumption (EPOC) after each short‐duration session (Børsheim and Bahr 2003; Laforgia et al. 2006). Despite this evidence, no previous study has compared in humans the effect of multiple short sessions of HIIE lasting 10 min or less throughout the day with a single HIIE session on EPOC.

Although the benefits of regular physical activity are well‐established, individuals often encounter challenges in adopting a physically active lifestyle, citing personal barriers related to perceived limitations in self‐efficacy and lack of time, among others. Thus, the use of short bouts, lasting less than 10 min, could be particularly advantageous for exercise adherence. Multiple shorter sessions might be perceived as less intense than a single session with higher volume, which could be crucial for self‐efficacy and the sustainability of an exercise program. However, it remains unclear whether multiple bouts of HIIE throughout the day are perceived as less intense and more achievable than a single session of HIIE. Additionally, practical limitations, such as hygiene issues, logistical challenges, and the need for appropriate scheduling, may influence the feasibility of implementing such protocols in real‐world settings.

In this study, we conducted a randomized crossover clinical trial involving middle‐aged individuals to compare the effects of a HIIE protocol performed in a single session versus three shorter sessions, with a 4 h interval between sessions, on EE, cardiometabolic responses, and perceived exertion. Our hypotheses were twofold: first, we expected that multiple sessions of HIIE would result in higher EPOC but lower cardiorespiratory response than a single session of HIIE. Second, we aimed to test whether three shorter sessions were perceived as intense as a single session of HIIE, hypothesizing that three shorter sessions of HIIE induce lower perceived exertion than a single session.

## 2 Methods

### 2.1 Ethical Considerations

This study was approved by the local institution's Ethics and Research Committee (protocol 60689122.9.00005108). Prior to participation, all individuals were informed of the study's objectives, procedures, potential risks, discomforts, and benefits. Each participant provided written informed consent, acknowledging their voluntary involvement and being free to withdraw from the study at any time.

### 2.2 Participants

Participants aged 45–64 years, who were nonsmokers, and categorized as recreationally physically active according to the International Physical Activity Questionnaire (IPAQ) were included in the study. Potential participants completed the Physical Activity Readiness Questionnaire (PAR‐Q), where any positive answer resulted in exclusion. Additionally, a medical questionnaire was administered, and the potential participant was excluded if diagnosed with cardiometabolic, renal, or pulmonary disease as well as any orthopedic or neurological limitations.

**FIGURE 1:**
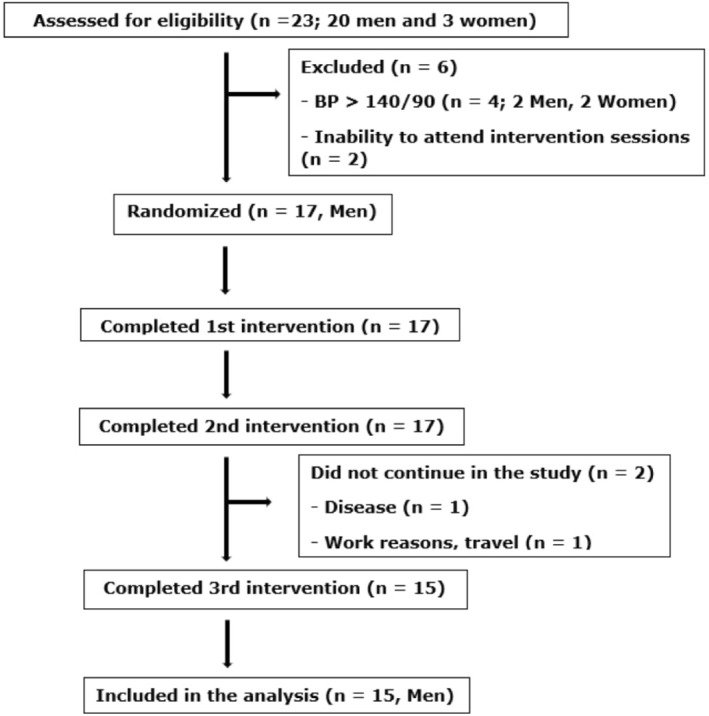
Flowchart of participants.

Twenty‐one individuals (18 men and 3 women) were recruited through flyers, social media platforms, and word of mouth. However, following the inclusion criteria described below, 15 men met the eligibility criteria and were invited to participate in the study. Although it was not the authors' intention to exclude women, two women did not meet the inclusion criteria and one was not able to attend all the experimental trials. Similarly, two males did not meet the inclusion criteria and one was not able to attend all the experimental trials. The remaining 15 participants completed all experimental procedures, and their data were included in the statistical analysis (Figure 1).

### 2.3 Study Design

**FIGURE 2:**
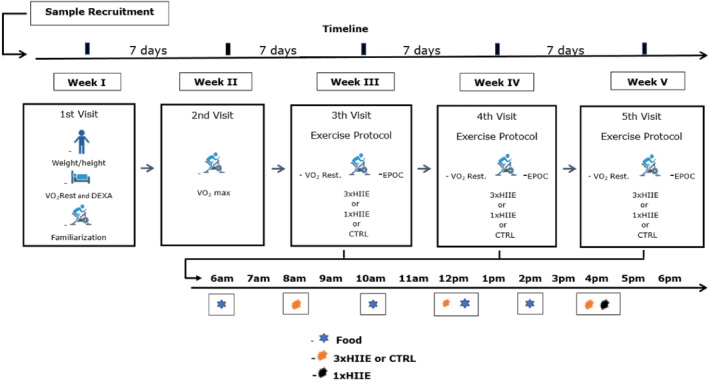
Experimental design.

This study employed a randomized crossover clinical trial design, registered in the Brazilian Registry of Clinical Trials (ReBEC) under the identifier RBR‐10kynbky. Participants underwent a total of five visits to the laboratory, totaling 2 baseline visits and 3 experimental sessions. Visits were separated by 7 days, with the first two used for baseline assessments. Subsequently, participants were randomly assigned to one of the three experimental sessions (Figure 2). During all visits, room temperature was maintained at 22°C and relative humidity was approximately 60%. Participants were instructed to wear similar clothing (shorts and t‐shirt) at all visits and advised to refrain from physical exercise and alcohol consumption for 48 h and caffeine for 12 h prior to each visit. Additionally, participants were instructed to wear an actigraph watch (wGT3X‐BT) for 48 h before each experimental session to record physical activity levels and sleep quantity and duration. They were also asked to follow a meal plan based on their food records for the 24 h before and during the day of each laboratory visit. Finally, they were advised to limit physical activity on the way to the laboratory to avoid an increase in metabolic rate.

### 2.4 Baseline Assessments

For all visits, participants arrived at the laboratory at 7:30 a.m. During visit 1, participants arrived after a 12 h overnight fast (Compher et al. 2006) for the measurement of resting oxygen consumption (V̇O_2_). Then, body composition was measured using dual‐energy x‐ray absorptiometry (DEXA – GE Healthcare, Madison, Wisconsin, EUA) followed by a standardized breakfast. Thirty minutes after breakfast, participants were familiarized with the maximal ramp protocol on a cycle ergometer. Thereafter, participants received instruction from a certified dietitian indicating how to use food log forms. They were instructed to complete the food logs for three nonconsecutive days (2 days during the week and one day on the weekend) prior to the second laboratory visit. Dietary intake was calculated as the average intake of the 3 days for total calories, carbohydrates, lipids, and protein. All dietary calculations were performed using a software (DietPro, version 5i, Viçosa, Minas Gerais/Brazil).

At the second visit, participants underwent a maximal ramp protocol (Myers and Bellin 2000) on a Lode cycle ergometer (Lode, Corival 400) to determine their V̇O_2_max. The ramp protocol consisted of a 3 min warm‐up at 30 W, followed by constant individualized increments in workload until fatigue despite verbal encouragement. The Lode cycle ergometer is equipped with a built‐in display that continuously shows the cadence. The test was performed at a cadence of 60 rpm. In addition, a researcher was present at all times to monitor whether the participant was maintaining the specified cadence throughout the exercise protocol. This dual monitoring approach ensured that the required cadence was maintained during the sessions. The criteria for achieving V̇O_2_max included meeting 2 of the following criteria: (i) a plateau in V̇O_2_ despite the increase in work rate; (ii) a respiratory exchange ratio (RER) greater than 1.10; (iii) a heart rate (HR) greater than 95% of the predicted maximal HR for the calculated age (220 – age); and (iv) volitional exhaustion as indicated by a perceived exertion score ≥ 18 on the Borg scale (Midgley et al. 2007).

All HIIE sessions were performed on the same Lode cycle ergometer in the laboratory. The maximum power output achieved during the VO_2_max test was used to prescribe the corresponding exercise intensities for the warm‐up, high‐intensity bouts, and active recovery phases of the exercise protocol. Specifically, for the initial warm‐up and recovery periods, the intensity was set at 30%–50% of maximal power, and for the high‐intensity bouts, the intensity was set at 90% of maximal power. This approach ensured that the prescribed intensities were accurately matched to each participant's individual capabilities as determined using their VO_2_max assessment. Following the test, participants were provided a meal plan based on the composition and total energy of their 3‐day food logs. This meal plan was to be followed in the 24 h before and during the days of the experimental sessions. Participants were instructed to consume a pre‐exercise meal consisting of 65% carbohydrates, 15% proteins, and 20% lipids 2 h before each experimental session.

### 2.5 Experimental Sessions

The three experimental sessions were previously randomly assigned using a number generator (https://www.randomizer.org/). At the arrival, participants were asked whether they adhered to the pre‐exercise recommendation, including abstaining from exercise and alcohol for the previous 48 h, from caffeine for the previous 12 h, following the meal plan, and limiting physical activity on the way to the laboratory. Actigraphy watches were then removed from participants to estimate physical activity levels and sleep quantity and duration in the 48 h prior to the experimental sessions. Then, participants sat quietly for 30 min before the start of the exercise session.

**FIGURE 3:**
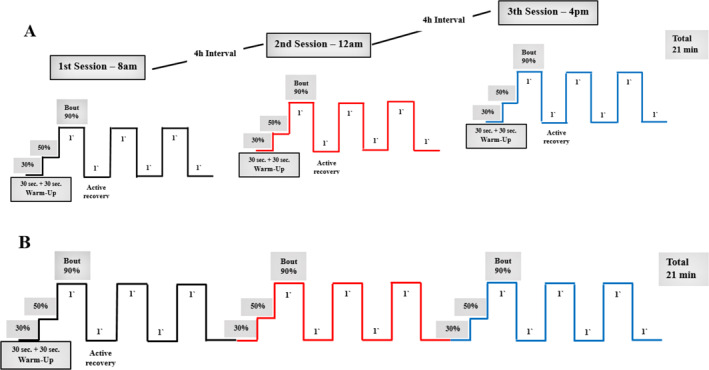
Exercise protocols.

The exercise sessions consisted of (i) a HIIE protocol performed in a single session (1xHIIE), (ii) a HIIE protocol performed in three shorter sessions with a 4 h interval between each session (3xHIIE), and (iii) a nonexercise control protocol (CTRL) (Figure 3). The exercise protocols were adapted from previous research (Matos et al. 2018).

- 3xHIIE

The 3xHIIE protocol consisted of three 7 min sessions (21 min total) with a 4 h interval between sessions starting at 8:00 a.m., 12:00 p.m., and 4:00 p.m. Each of these 7 min sessions consisted of a 1 min warm‐up (consisting of 30 s at 30% and 30 s at 50% of V̇O_2_max), followed by three 1 min bouts at 90% of V̇O_2_max, with 1 min periods of active recovery at 30% of V̇O_2_max after each bout (Figure 3A).

- 1xHIIE

The 1xHIIE protocol session maintained the same volume and intensity as the 3xHIIE protocol, except that participant performed in a single 21 min the HIIE session (Figure 3B). The 1xHIIE protocol was designed to end at 4:30 p.m., to coincide with the end of the third session of the 3xHIIE protocol. This strategy was used to minimize potential interferences from the circadian cycle and feeding schedule.

- CTRL protocol

The CTRL protocol was conducted to minimize interference from food intake and the circadian cycle. During the nonexercise control protocol, procedures were identical to the HIIE‐3X protocol, except that the participant remained quietly seated in a chair without engaging in any exercise.

## 3 Physiological and Perceptual Measurements

### 3.1 Pre‐Exercise Measures

Upon arrival at the laboratory, participants were asked to sit quietly in a chair for 30 min. During this time, they wore a face mask connected to a gas analyzer (Lab Chart Pro Metabolic Module V8 and AD Metabolic System Instrument PowerLab #DM‐060‐24) for breath‐by‐breath measurements. In addition, participants wore an HR monitor (Polar RS800 HR Electro, Finland). They were instructed to remain motionless and refrain from using their phones or engaging in any other activities to prevent potential changes in V̇O_2_. Resting V̇O_2_ and HR were calculated using the last 5 min of the pre‐exercise period. The lowest average of five consecutive 1 min measurements was used as baseline V̇O_2_ values (Compher et al. 2006; Valstad et al. 2018). Resting blood lactate was obtained from a fingertip blood sample during the last minute of the resting period using a portable lactate analyzer (Accutrend Plus, D‐68298 Mannheim, Germany, Roche Diagnostics). The Accutrend Plus was validated for use in the field of sports research in a previous study (Baldari et al. 2009).

### 3.2 Exercise Measurements

The gas analyzer displays the results of gas exchange every 10 s, and the average of every six measurements was taken to determine VO_2_ from minute to minute. Heart rate was recorded before exercise at the fifth minute of the VO_2_ recording. During exercise, HR was recorded each 1 min. After exercise, HR was recorded each 5 min for 15. The rating of perceived exertion was assessed immediately after each interval, and blood lactate levels were recorded immediately after each exercise session.

### 3.3 Postexercise Measures

Immediately after exercise, participants sat in a chair until their V̇O_2_ levels returned to baseline V̇O_2_ values. Two methods were used to determine the termination of EPOC. The first method—the 1 standard deviation method—identified EPOC termination as the time when V̇O_2_ values fell within 1 standard deviation of baseline V̇O_2_ for two consecutive minutes (Dawson et al. 1996; Chad and Wenger 1988). The second method—the 5 min EPOC method—identified the end of EPOC as the time when the 5 min average V̇O_2_ equaled baseline V̇O_2_ (Valstad et al. 2018; Sedlock 1994; Sedlock et al. 1989). For both methods, the duration from the end of the exercise to the end of EPOC was considered the EPOC duration (Short and Sedlock 1997). EPOC was calculated as the difference between postexercise V̇O_2_ and resting V̇O_2_ (Valstad et al. 2018; Matthews et al. 2022; McGarvey et al. 2005).

For the 3xHIIE protocol, EPOC was calculated as the sum of V̇O_2_ during the recovery periods following the three exercise sessions, whereas HR and blood lactate were averaged across these recovery periods. Energy expenditure (in kcal) was calculated by multiplying the total VO_2_ (L) by 5 kcal/L as described previously (Matthews et al. 2022; Tucker et al. 2016). For the nonexercise (CTRL) protocol, V̇O_2_ and HR were recorded identically to the 3xHIIE protocol.

## 4 Statistical Analysis

Data are presented as mean ± standard deviation. A priori sample size calculation was performed using the G*Power 3.1 software, with an alpha value of 0.05% and 80% power. The calculation was based on detecting a moderate effect size (Cohen's *f* = 0.25) for the interaction effect and two‐way repeated measures ANOVA (3 conditions × 3‐time points), assuming a correlation of 0.5 between repeated measures. This calculation was based on a previous study that found significant differences in EPOC between two exercises and CTRL protocols (Islam et al. 2018), resulting in a minimum sample size of 12 participants. To account for the potential dropout of volunteers, an additional 20% was added to the sample size, resulting in a final sample size of 15 individuals. The normality of the data was assessed using the Shapiro–Wilk test. One‐way ANOVA and Tukey’s post hoc test were used to compare the total energy expenditure and EPOC. Two‐way analysis of variance with repeated measures and Tukey’s post hoc test were used to investigate differences in VO_2_, HR, RPE, and blood lactate responses. Student's *t*‐test was used to compare exercise and recovery energy expenditure between 1xHIIE and 3xHIIE protocols. The effect size was calculated using eta‐squared (*η* ^2^) for main effects and interactions in ANOVA and Hedge's *g* with 95% confidence intervals for paired post hoc contrasts. A significance level of 5% was used to determine statistical significance. Analyses were performed using GraphPad Prism, version 8.0, and Gpower 3.1.9.2.

## 5 Results

Table 1 displays the characteristics of the participants, including age, physical characteristics, V̇O_2_max, estimated resting metabolic rate, and peak power output during the maximal exercise test. Table 2 presents data on sleep quantity and markers of sleep quality the night preceding the experimental sessions as well as physical activity levels 48 h before the experimental sessions. No differences were observed among the experimental sessions in any of these parameters.

**TABLE 1:** Participant characteristics (*N* = 15).

	Mean	± SD
Age (yr)	48.53	2.97
Height (m)	1.74	0.06
Body mass (kg)	79.19	13.17
BMI (kg/m^2^)	25.92	3.09
% Body fat	18.07	13.18
% Lean body mass	57.85	8.74
RMR (Kcal)	1434	177.23
VO_2max_ (mL.Kg^−1^.min^−1^)	48.24	8.41
PPO (W)	285.7	47.88

*Note:* Data are presented as mean ± SD.

Abbreviations: BMI: body mass index; PPO: peak power output; RMR: resting metabolic rate; V̇O2max: maximal oxygen consumption.

**TABLE 2:** Physical activity levels 48 h before the experimental protocols and sleep quantity and quality the night before the experimental protocols.

	CTRL	1xHIIE	3xHIIE	*p*	F
Nº of steps 48 h pre	6711 ± 1822	7047 ± 1741	5608 ± 2799	0.200	(2, 39) = 1.677
Sleep:					
Total sleep time (min)	370 ± 31	350 ± 31	359 ± 38	0.310	(2, 36) = 1.210
Nº awakenings	22 ± 7.3	21 ± 6	22 ± 9	0.869	(2, 36) = 0.140
Awakenings (min)	59 ± 25.5	56 ± 15.2	61 ± 26.3	0.857	(2, 36) = 0.155
Mobility index	13.75 ± 4.4	14.3 ± 3.3	15 ± 4.2	0.723	(2, 36) = 0.326
Fragmentation index	23.6 ± 7.5	28.4 ± 6.3	28.4 ± 9	0.195	(2, 36) = 1.707
Efficiency (%)	86.4 ± 6.3	86.5 ± 3.3	85.0 ± 6	0.748	(2, 36) = 0.292
Latency	22 ± 16.6	15.4 ± 10.5	19.6 ± 12.6	0.455	(2, 36) = 0.804
Total time in bed (min)	7.40 ± 1.18	7.59 ± 0.46	7.62 ± 1.20	0.829	(2, 27) = 0.188

*Note:* Data are presented as mean ± SD, standard deviation. ANOVA one way, *p* < 0.05.

Abbreviations: 1xHIIE: high‐intensity interval exercise protocol performed in a single session; 3xHIIE: high‐intensity interval exercise protocol performed in three shorter sessions with a 4 h interval between sessions; CTRL: nonexercise control protocol.

**FIGURE 4:**
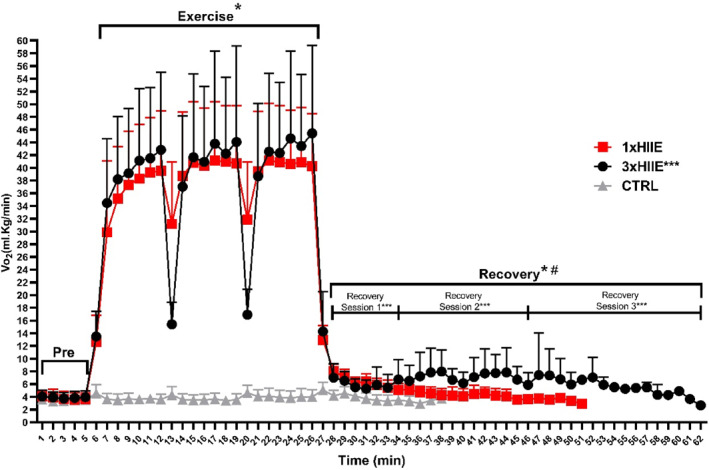
Oxygen consumption behavior. CTRL: control situation gray line. 1xHIIE: single session high‐intensity interval exercise red line. 3xHIIE: high‐intensity interval exercise 3 sessions black line. Data are presented as mean ± SD, standard deviation; two‐way ANOVA followed using Tukey's test, *n* = 15. *difference from CTRL. ^#^difference between 1xHIIE and 3xHIIE. ***Recovery period of sessions 1, 2, and 3 of the 3xHIIE protocol.

Figure 4 provides a representative illustration of the VO_2_ before, during, and after exercise. Data analysis was carried out separately for the pre‐exercise, exercise, and postexercise periods. Resting V̇O_2_ was similar across the CTRL, 1xHIIE, and 3xHIIE protocols, with no significant interaction between time and protocol F (8, 210) = 0.108, *P* = 0.99, and *η* ^2^ = 0.0041. During exercise, a significant interaction between time and protocol was observed for V̇O_2_ F (40, 882) = 6.459, *p* < 0.0001, and *η* ^2^ = 0.227, indicating that V̇O_2_ responses differed across time points and protocols. Additionally, significant main effects were found for time F (20, 882) = 16.87, *p* < 0.0001, and *η* ^2^ = 0.278 and protocol F (2, 882) = 1730, *p* < 0.0001, and *η* ^2^ = 0.797. There was a significant increase in VO_2_ from rest to exercise in both exercise protocols (1xHIIE: 2.86 ± 0.65 L/min vs. 0.30 ± 0.06 L/min, *p* < 0.0001 and 3xHIIE: 2.81 ± 0.47 L/min vs. 0.28 ± 0.07 L/min, *p* < 0.001). During exercise, V̇O_2_ was similar between the 1xHIIE and 3xHIIE sessions (*p* = 0.8915). During the recovery phase, an interaction between time and protocol was observed F (2, 28) = 4019, *p* < 0.0001, and *η* ^2^ = 0.741, with V̇O_2_ being significantly higher in the 3xHIIE protocol compared to the 1xHIIE protocol (*p* < 0.0001) and both exercise protocols exhibiting higher V̇O_2_ than CTRL.

EE and EPOC data are shown in Table 3. Total EE was significantly greater after the 3xHIIE protocol compared with the 1xHIIE protocol (362.28 ± 73.95 kcal vs. 325.62 ± 54.82 kcal, *t* = 4.867, *g* = 0.55, 95% CI [−0.18–1.28], and *p* = 0.0002). This effect was attributed to a greater EE during the exercise recovery phase of the 3 × HIIE protocol compared to the 1 × HIIE protocol (62.97 ± 14.97 vs. 27.42 ± 8.90 kcal, *t* = 9.782, *g =* 2.82, 95% CI [1.78–3.84], and *p* < 0.0001), given that EE during exercise was similar between the 3xHIIE and 1xHIIE protocols (299.32 ± 69.18 vs. 298.20 ± 51.74, *t* = 0.145, *g =* 0.01, 95% CI [−0.6–0.73], and *p* = 0.88). EPOC was significantly higher in the 3xHIIE protocol compared to the 1xHIIE protocol (5.70 ± 2.11 vs. 2.35 ± 1,09 LO_2_, *t* = 6.606, *g =* 1.94, 95% CI [1.06–2.82], and *p* < 0.0001). Moreover, the duration of EPOC was also significantly higher in the 3xHIIE protocol compared to the 1xHIIE protocol (23:23 ± 04:45 vs. 11:47 ± 04:07 min, *t* = 7.013, *g =* 2.69, 95% CI [1.69–3.69], and *p* < 0.0001).

**TABLE 3:** VO_2_ oxygen during the experimental protocols.

	1xHIIE		3xHIIE				
	Mean	± SD	Mean	± SD	*p*	T	Effect size/power
VO_2_ rest (L/min^−1^)	0.28	0.07	0.30	0.06	0.29	1.08	
VO_2_ total exercise (LO_2_)	53.61	9.09	53.56	13.19	0.96	0.04	
E.E. exercise (Kcal)	298.20	51.74	299.32	69.18	0.88	0.14	
E.E. recovery (Kcal)	27.42	8.90	62.97^#^	14.97	0.0001	9.78	2.72/1
T.E.E. total (Kcal)	325.62	54.82	362.28^#^	73.95	0.0002	4.86	0.55/0.83
EPOC (LO_2_)	2.35	1.09	5.70^#^	2.11	0.0001	6.60	1.83/1
EPOC (Kcal)	11.76	5.45	28.50^#^	10.57	0.0001	6.60	1.83/1
EPOC magnitude (min)	11:47	04:07	23:23^#^	04:45	0.0001	7.01	2.75/1

*Note:* Data are presented as mean ± SD, standard deviation. Test *T Student;* ^#^ difference between 1xHIIE and 3xHIIE. *p* < 0.05.

Abbreviations: 1xHIIE: high‐intensity interval exercise protocol performed in a single session; 3xHIIE: high‐intensity interval exercise protocol performed in three shorter sessions with a 4 h interval between sessions; E.E.: energy expenditure in kilocalories; EPOC: excess postexercise oxygen consumption; VO2: oxygen consumption.

**FIGURE 5:**
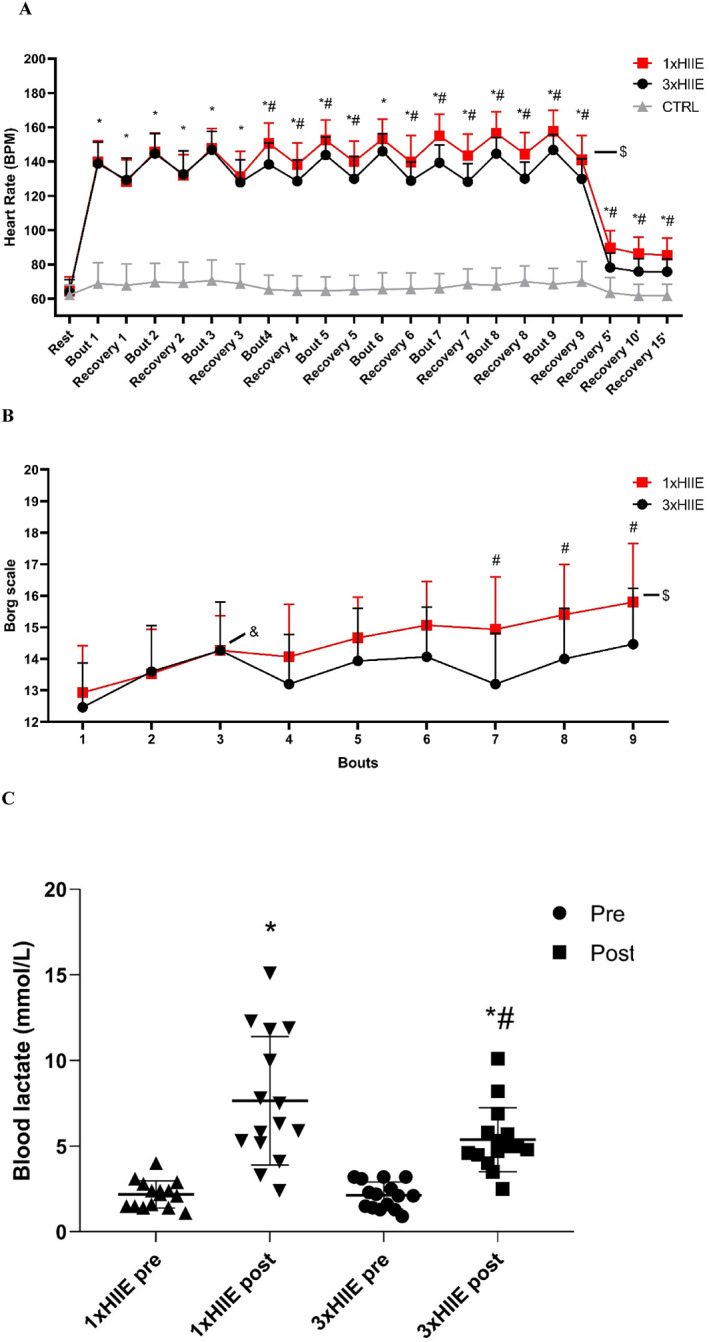
Heart rate during the experimental protocols (A), subjective perception of effort (B), and blood lactate (C). CTRL: control situation protocol. 1xHIIE: high‐intensity interval exercise protocol performed in a single session. 3xHIIE: high‐intensity interval exercise protocol performed in three shorter sessions with a 4 h interval between sessions. Data are presented as mean ± SD, standard deviation. (A,B) ANOVA two‐way followed using Tukey's test. (C) ANOVA one‐way followed using Tukey's test. *difference from CTRL; ^#^difference between 1xHIIE and 3xHIIE; ^$^difference between Bout 1 1xHIIE and Bout 9 1xHIIE;^$^difference between Bout 1 1xHIIE and Bout 9 1xHIIE; ^&^difference between Bout 1 3xHIIE and Bout 3. *difference between pre and post; ^#^difference between 1xHIIE and 3xHIIE. *p* < 0.05. HR, effect size = 0.65 and power 0.99; RPE, effect size 0.59 and power 0.88; and lactate, effect size = 0.69 and power 0.95.

Figure 5A illustrates heart rate (HR) measurements before, during, and after exercise. A significant time and protocol interaction was observed for HR F (42, 924) = 25.24, *p* < 0.0001, and *η* ^2^ 0.534, indicating that HR responses differed across time points and exercise protocols. Additionally, significant main effects were observed for time F (21, 924) = 121.8, *p* < 0.0001, and *η* ^2^ 0.735 and protocol F (2, 924) = 3605, *p* < 0.0001, and *η* ^2^ = 0.886. Resting HR was similar among the CTRL, 1xHIIE, and 3xHIIE protocols (CTRL: 62 ± 5.9 bpm vs. 1xHIIE: 64 ± 7.3 bpm and *p* = 0.8546; CTRL: 62 ± 5.9 bpm vs. 3xHIIE: 64 ± 8.2 bpm and *p* = 0.9085; and 1xHIIE: 64 ± 7.3 bpm vs. 3xHIIE: 64 ± 8.2 bpm and *p* = 0.9925). The CTRL protocol showed no significant HR changes throughout the recording period (*p* > 0.9999). Post hoc analysis revealed a significant increase in HR from rest to exercise in both exercise protocols (1xHIIE: from 64 ± 8.28 bpm to 140 ± 12.3 bpm, *p* < 0.001 and 3xHIIE: from 64 ± 7.27 bpm to 139 ± 12.5 bpm, *p* < 0.0001). During exercise, HR was significantly higher in the 1xHIIE protocol compared to the 3xHIIE protocol from the fourth bout to the end of the session (*p* < 0.05). Within the 1xHIIE protocol, HR during the eighth and ninth bouts was significantly higher than in the first bout (*p* = 0.04 and *p* = 0.01, respectively). During the recovery period, HR remained higher in the 1xHIIE protocol compared to the 3xHIIE protocol at five minutes (*p* = 0.01), 10 minutes (*p* = 0.02), and 15 minutes (*p* = 0.04).

Figure 5B shows RPE responses to the 1xHIIE and 3xHIIE protocols. A significant time and protocol interaction was observed for RPE, F (8, 112) = 4.734, *p* < 0.0001, and *η* ^2^ 0.253, indicating that RPE responses differed across time points and protocols. Additionally, significant main effects were observed for time F (8, 112) = 29.49, *p* < 0.0001, and *η* ^2^ 0.678 and protocol F (1, 14) = 11.26, *p* = 0.0047, and *η* ^2^ 0.446. The 1xHIIE protocol showed a progressive increase in RPE from the second to the ninth bout (*p* < 0.0001). In contrast, the 3xHIIE protocol exhibited a progressive increase in RPE from the first to the third bout (*p* < 0.0001) and from the seventh to the ninth bout (*p* = 0.003), corresponding to the last bout of each of the three sessions. No significant difference in RPE was observed among the three sessions of the 3xHIIE protocol (*p* > 0.9999). When comparing RPE between the 1xHIIE and 3xHIIE protocols (considering a total of 9 bouts), RPE in the 1xHIIE protocol was significantly higher than in the 3xHIIE protocol from the seventh to the ninth bout (*p* < 0.05).

Blood lactate responses are presented in Figure 5C. The mean blood lactate immediately after each of the three 3xHIIE protocol sessions was significantly lower than the blood lactate immediately after the 1xHIIE protocol session F (3, 42) = 26.85, *p* < 0.0001, and *η* ^2^ = 0.657.

## 6 Discussion

The purpose of this study was to compare the acute effects of an HIIE protocol performed either in a single longer session (1xHIIE) or in three shorter sessions (3xHIIE) on EPOC, EE, cardiometabolic responses, and perceived exertion. The main findings were that partitioning the HIIE protocol into three 7 min sessions yields higher total EE with less perceived exertion compared to a single 21 min session. As expected, the 3xHIIE and 1xHIIE protocols induced similar EE during exercise, but EE during the recovery period was nearly twice as high in the 3xHIIE protocol compared to the 1xHIIE protocol. Furthermore, HR, blood lactate levels, and RPE responses were lower during the 3xHIIE compared to the 1xHIIE protocol. These findings provide evidence for an effective way to increase total energy expenditure with less perceived effort when HIIE is spread over multiple short bouts.

Confirming our hypothesis, EPOC was significantly higher in the 3xHIIE protocol compared to the 1xHIIE protocol, despite similar V̇O_2_ during the exercise protocols. Consequently, the 3xHIIE protocol resulted in a significantly higher calculated energy expenditure, approximately 40 kcal greater than that of the 1xHIIE protocol. Although some may argue that a 40 kcal difference in energy expenditure between the 3xHIIE and 1xHIIE protocols might not hold clinical significance, empirical evidence suggests otherwise. A comprehensive study using data from national surveys conducted between 1988 and 2000 revealed that a mere daily calorie deficit of 50 kcal could prevent weight gain in 90% of adults (Hill et al. 2003). Thus, extrapolating the superior daily energy expenditure of approximately 40 kcal from the 3xHIIE protocol over several weeks, months, or years could lead to a significantly higher cumulative energy expenditure. This cumulative effect has the potential for a significant impact on bodyweight management, particularly among middle‐aged individuals.

In our previous laboratory animal studies, we demonstrated that both MICT (moderate‐intensity continuous training) (Costa‐Pereira et al. 2017) and HIIT (Mendes et al. 2022) protocols, when performed with three shorter sessions throughout the day, resulted in more significant reductions in body fat, visceral fat, and adipocyte size compared to the same exercise training protocols performed in a single longer daily session. These significant reductions in body fat, visceral fat, and adipocyte size observed in our study occurred irrespective of the calorie intake of the animals.

Studies have shown that HIIT results in greater total energy expenditure (EE) and EPOC compared to moderate‐intensity continuous training (MICT) even when EE during exercise is matched (Jiang et al. 2024). This suggests that higher exercise intensity is more important than duration in generating EPOC. Furthermore, interval training patterns may be more favorable for EPOC production than continuous training due to their ability to induce repeated metabolic peaks (Tucker et al. 2016; Jiang et al. 2024). Murphy (Murphy et al. 2019) speculated that small increases in metabolism occurring following two or more daily sessions of exercise may lead to a higher EPOC magnitude compared to a single daily exercise session. The higher EPOC observed following multiple versus single daily sessions of high‐intensity exercise can be attributed to some key mechanisms. First, due to its intense nature, each session of HIIT induces significant metabolic disturbances, requiring substantial energy expenditure for recovery processes such as muscle repair and replenishment of energy stores. When these intense workouts are repeated throughout the day, they may result in a greater overall demand for oxygen after exercise. Therefore, incorporating multiple HIIT sessions into a daily routine may be an effective strategy for increasing EPOC, EE, and improving weight loss. Although in the present study, we measured this response for a single day (acute effect), we speculate that it may be beneficial in reducing body fat over a prolonged period (chronic effect). Further studies conducted in humans are warranted to investigate the long‐term effects of this response during an exercise‐training program.

In addition to the higher EPOC, the 3xHIIE protocol elicited lower acute cardiometabolic responses and perceived exertion compared to the 1xHIIE, despite the same workload and duration including the warm‐up and active recovery. This is evidenced by lower HR, blood lactate levels, and RPE in response to the 3xHIIE protocol compared to the 1xHIIE.

A lower cardiovascular response during high‐intensity exercise may improve safety for both well‐trained individuals and those with cardiovascular disease (Ha et al. 2002; Huang et al. 2008). For example, an exaggerated systolic blood pressure response during exercise (≥ 210 mmHg for men and ≥ 190 mmHg for women) is considered abnormal and may signal an increased risk of future cardiovascular events (Le et al. 2008). Similarly, an increase in diastolic blood pressure of more than 10 mmHg above resting levels, or an absolute value of 90 mmHg, is also considered abnormal (Ha et al. 2002). Therefore, the 3xHIIE protocol, which elicits a milder cardiovascular response than the 1xHIIE, may be particularly beneficial for individuals with cardiometabolic conditions, such as hypertension, heart disease, and diabetes, as it may promote safer exercise practices while still providing effective training benefits.

The observed differences in heart rate responses between the two protocols may be partly explained by the exercise duration. In the single 21 min session (1xHIIT), participants performed the exercise continuously, which is likely to have resulted in a gradual increase in heart rate over time due to fatigue and other factors such as dehydration or heat build‐up. In contrast, the three shorter 7 min sessions with 4 h intervals (3xHIIT) allowed for partial recovery between bouts, which may result in a lower heart rate despite similar oxygen consumption (VO_2_) between protocols. This suggests that the 4 h breaks between sessions in the 3xHIIT protocol mitigated the gradual increase in heart rate typically associated with prolonged exercise.

It is important to acknowledge the limitations of the present study. The small sample size of this study is acknowledged as a limitation. Our results are applicable to recreationally active middle‐aged men. Although using recreationally active individuals can be seen as a limitation, we would like to highlight that independent of physical activity levels, sedentary behavior increases the risk for several diseases such as cardiovascular diseases (Rezende et al. 2014) and type 2 diabetes mellitus (Wilmot et al. 2012). Since we used calculations for the energy expenditure results, it may be underestimated or overestimated; however, the identical calculation for both HIIT protocols ensures the higher energy expenditure of the 3xHIIT protocol compared to the 1xHIIT protocol. Additionally, we employed two methods to establish the termination of EPOC as criteria for interrupting the V̇O_2_ during exercise recovery. These methods recommend discontinuing the recording after V̇O_2_ returns to resting values for 5 consecutive minutes and when V̇O_2_ values fall within 1 standard deviation of baseline V̇O_2_ for two consecutive minutes. Using these established criteria, EPOC in the current study lasted 7–35 min. Therefore, we cannot rule out the possibility of a lingering effect on EPOC after this period nor can we know whether differences existed between HIIE protocols. Thus, future studies in this area should focus on measuring EPOC for greater periods (e.g., > 1 h) to gain a better understanding of the EPOC in response to the 1xHIIE and 3xHIIE protocols.

The present study also has multiple strengths. First, this is the first randomized crossover clinical trial to compare the EE and cardiometabolic responses using a single session versus three shorter sessions of an HIIE protocol. Secondly, we carefully monitored several potential confounding factors that could interfere with the study's outcomes, including sleep quantity and quality, food consumption, and physical activity levels. It is important to emphasize that all these variables were recorded 24–48 days before the experimental sessions as well as in the experimental days, and it was observed no differences in these variables among protocols. Finally, we emphasize the addition of the CTRL no‐exercise protocol, which was conducted at the same hours of the day as the 3xHIIE sessions, to minimize potential interference from circadian rhythms and food intake. Finally, the accumulated HIIE protocol seems to be a prominent strategy for enhancing cardiovascular health, since HIIT protocols lasting less than 15 min can still yield cardiometabolic benefits in the general population (Coates et al. 2023).

An important practical consideration is the adherence of the general population to multiple short daily exercise sessions. Practical constraints, such as the need for multiple showers and changes of clothing throughout the day, may affect individuals' willingness to commit to this exercise program in the long term. However, we believe that exercise sessions of less than 2–3 min several times a day may be effective, and individuals may not need to shower and change clothes after each exercise session. Another barrier may be the need for access to specialized equipment throughout the day. However, studies have shown that adherence can be achieved with exercises that do not require specialized equipment, such as bodyweight exercises (Sharp et al. 2024), stair climbing (Stork et al. 2024), or even small cycle ergometers at the workplace (Kakarot and Müller 2014).

Thus, from a public health perspective, beyond practicing an exercise with higher energy expenditure and lower effort, as we saw in the current study, these small adaptations may be crucial for increasing adherence to multiple short daily exercise sessions by the general population.

## 7 Conclusion

In summary, our findings collectively demonstrate that partitioning an HIIE into shorter bouts throughout the day results in higher energy expenditure with reduced cardiometabolic and perceived exertion in middle‐aged individuals. Therefore, multiple bouts of HIIE can facilitate body weight management as well as reduce sedentary behavior.

## 8 Perspectives

Future studies should examine the long‐term effects of multiple versus single daily sessions of HIIE on energy expenditure and weight loss. The results of this study suggest that multiple shorter sessions of HIIE throughout the day (3xHIIE) may be a more effective approach than single longer daily sessions of HIIE (1xHIIE) for exercise programs aimed at preventing weight gain and obesity. By incorporating multiple short sessions of HIIE throughout the day, individuals may achieve higher overall energy expenditure with less perceived exertion, potentially increasing adherence to exercise programs and improving long‐term health outcomes.

## Ethics Statement

This study was approved by the local institution's Ethics and Research Committee (protocol 60689122.9.00005108).

## Conflicts of Interest

The authors declare no conflicts of interest.
